# Enhancing Long-Term Durability of Electrochemical Reactors Producing Formate from CO_2_ and Water Designed for Integration with Solar Cells

**DOI:** 10.1021/acsomega.3c08911

**Published:** 2024-03-01

**Authors:** Naohiko Kato, Yasuaki Kawai, Natsumi Nojiri, Masahito Shiozawa, Yoshihiro Kikuzawa, Nobuaki Suzuki, Satoru Kosaka, Yuichi Kato, Juntaro Seki, Tsuyoshi Hamaguchi, Yasuhiko Takeda

**Affiliations:** Toyota Central R&D Labs., Inc., Nagakute, Aichi 480-1192, Japan

## Abstract

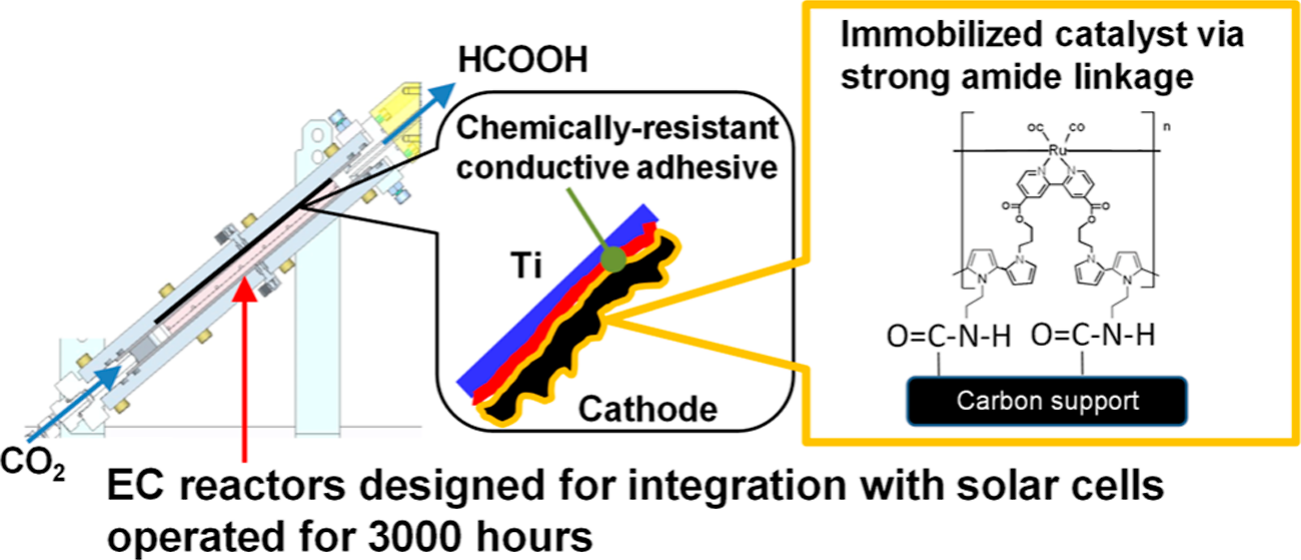

Artificial photosynthetic cells producing organic matter from CO_2_ and water have been extensively studied for carbon neutrality, and the research trend is currently transitioning from proof of concept using small-sized cells to large-scale demonstrations for practical applications. We previously demonstrated a 1 m^2^ size cell in which an electrochemical (EC) reactor featuring a ruthenium (Ru)-complex polymer (RuCP) cathode catalyst was integrated with photovoltaic cells. In this study, we tackled the remaining issue to improve the long-term durability of cathode electrodes used in the EC reactors, demonstrating high Faradaic efficiencies exceeding 80% and around 60% electricity-to-chemical energy-conversion efficiencies of a 75 cm^2^ sized EC reactor after continuous operation for 3000 h under practical conditions. Introduction of a pyrrole derivative containing an amino group in the RuCP coupled with UV–ozone treatment to create carboxyl groups on the carbon supports effectively reduced the detachment of the RuCP catalyst by forming a strong amide linkage. A newly developed chemically resistant graphite adhesive prevented the carbon supports from peeling off of the conductive substrates. In addition, highly durable anodes composed of IrO_*x*_-TaO_*y*_/Pt-metal oxide/Ti were adopted. Even though the EC reactor was installed at an inclined angle of 30°, which is approximately the optimal angle for receiving more solar energy, the crossover reactions were sufficiently suppressed because the porous separator film impeded the transfer of oxygen gas bubbles from the anode to the cathode. The intermittent operation improved the energy-conversion efficiency because the accumulated bubbles were removed at night.

## Introduction

Artificial photosynthesis, in other words, conversion of CO_2_ and water into valuable organic matter using solar energy, comprises two aspects: (I) fixation of CO_2_ and substantial reduction of CO_2_ emission and (II) storage of solar energy. Consequently, this field has witnessed extensive research and development in recent years,^1−5^ with the current research trend transitioning from proof of concepts on the basis of catalyst materials and small-sized cells to large-scale demonstrations for practical applications. Notably, experimental demonstrations involving 100 m^2^ scale photocatalyst panels for hydrogen gas (H_2_)-production are underway.^6^ On the other hand, integration of electrochemical (EC) reactors and photovoltaic (PV) cells has proceeded for the CO_2_ reduction reaction (CO2RR). For instance, one of the largest artificial photosynthetic cells adopted the EC–PV integration using EC reactors with an indium cathode catalyst to produce formate and crystalline-silicon solar cells with an irradiation area of 1.5 m^2^.^7^ Formic acid holds promise as a feed preservative, a raw material for chemical products,^8−10^ and an H_2_ carrier, as well as their use in direct formic-acid fuel cells.^11−13^ However, the conversion efficiency from the solar energy to the chemical energy (*η*_STC_) was as low as 1.9%, primarily due to a high overvoltage.^7^ Other conventional metal and metal-oxide catalysts employed in the CO2RR have also encountered similar difficulties. Therefore, improvements in *η*_STC_ by reducing the overvoltage and the overall efficiency of artificial photosynthesis are strongly needed.

In our previous work, we successfully developed a molecular catalyst composed of a ruthenium (Ru)-complex polymer (RuCP) to produce formate.^14^ The cathode electrodes comprising the RuCP catalyst loaded on carbon supports consisting of carbon-fiber sheets (CSs) coated with multiwalled carbon nanotubes (MWCNTs) bonded to titanium (Ti) plates, referred to as RuCP/MWCNTs/CS/Ti-plates hereinafter, demonstrated impressively low overvoltages (e.g., 0.02 V at 0.9 mA/cm^2^).^15−17^ Artificial photosynthetic cells of monolithic type with a 1 cm^2^ active area, modeled after a plant and referred to as an “artificial leaf,” achieved a *η*_STC_ of 4.3–4.6% using the RuCP-based cathodes and iridium oxide (IrO_*x*_)-based anodes.^18,19^ Based on this technology, we tackled the challenges unique to scale-up of artificial photosynthetic cells. We fabricated 1000 cm^2^ size cells of EC–PV integration with a higher *η*_STC_ of 7.2%.^16^ Furthermore, we realized an even higher *η*_STC_ of 10.5%, employing larger anodes and cathodes measuring 1 m^2^.^17^ For practical applications, cost-effective materials and manufacturing processes are of critical importance in addition to securing high *η*_STC_ and durability. Therefore, we adopted crystalline-silicon solar cells for integration with the EC reactors that are more economical than GaAs-based tandem solar cells often used to achieve high *η*_STC_.^20,21^ Furthermore, instead of a two-chamber reactor using an ion-exchange membrane inserted between the anode and the cathode, which added to the costs, we employed a single-chamber reactor and a porous separator. This type of reactor often raises a serious issue of crossover reactions. However, the RuCP catalyst effectively suppressed the oxygen reduction reaction (ORR), while the IrO_*x*_ anode catalyst used in the reactors was less active to oxidation of the produced formate. Further, the porous separator prevented the oxygen gas (O_2_) bubbles generated on the anode from transferring to the cathode, thus suppressing the ORR. As a result, we eliminated these crossover reactions, resulting in high Faradaic efficiencies (FEs) over a broad range of operating voltages.^16^ Another development unique to scale-up included stacked anode and cathode electrodes employing Ti plates with low electric resistance to match the charge generation rate of solar cells and the reaction rate of the EC reactor. Additionally, we incorporated well-designed electrolyte-flow channels in the reactor housing to guarantee the uniform supply of CO_2_ dissolved in the electrolyte.^17^

In the present study, we addressed the two remaining crucial issues concerning the practical applications of large-sized EC–PV cells with high *η*_STC_. The first challenge revolved around ensuring long-term durability. The operating current of the 1000 cm^2^ size EC reactor gradually decreased during continuous operation for dozens of hours. We meticulously investigated the causes of this degradation and identified three key factors, which are described in detail later. In brief, two of the factors were associated with the cathode electrodes: the peeling off of the carbon supports from the Ti plates and detachment of the RuCP catalyst from the carbon supports. The third factor involved the detachment of the anode catalyst, that is, IrO_*x*_ particles, from the Ti plates.^22^

Another critical issue we addressed was the validity of laboratory-based evaluations for reflecting outdoor operations. In our previous work, we vertically installed the integrated EC–PV cells on the ground and evaluated their performance under continuous simulated sunlight (ca. 1 sun).^16,17^ This vertical setup was chosen to prevent a decrease in *η*_STC_ caused by the crossover reactions that would occur more notably when the EC–PV cells are inclined. Indeed, it has been reported that the ORR of the O_2_ bubbles generated on the anode and transferred to the cathode can be substantial in a single-chamber water-splitting reactor lacking a separator.^23^ However, an inclined installation is more advantageous than the vertical installation for receiving more solar energy, given that sunlight shines from inclined directions, similar to solar cells.^24,25^ In this study, we found that the porous separator effectively suppressed the crossover reactions, including the ORR, even when the EC reactor for the CO2RR was installed at an inclined angle. Subsequently, we conducted a long-term durability test for 3000 h on an electrolyte-flow-type EC reactor. This system featured an active area of 5 cm × 15 cm and was positioned at an inclined angle of 30° using the electrolyte-circulating equipment.

## Results and Discussion

### Improvements in Adhesion between the RuCP/MWCNTs/CSs and Ti Plates Using the Novel Graphite Adhesive

**Figure 1:**
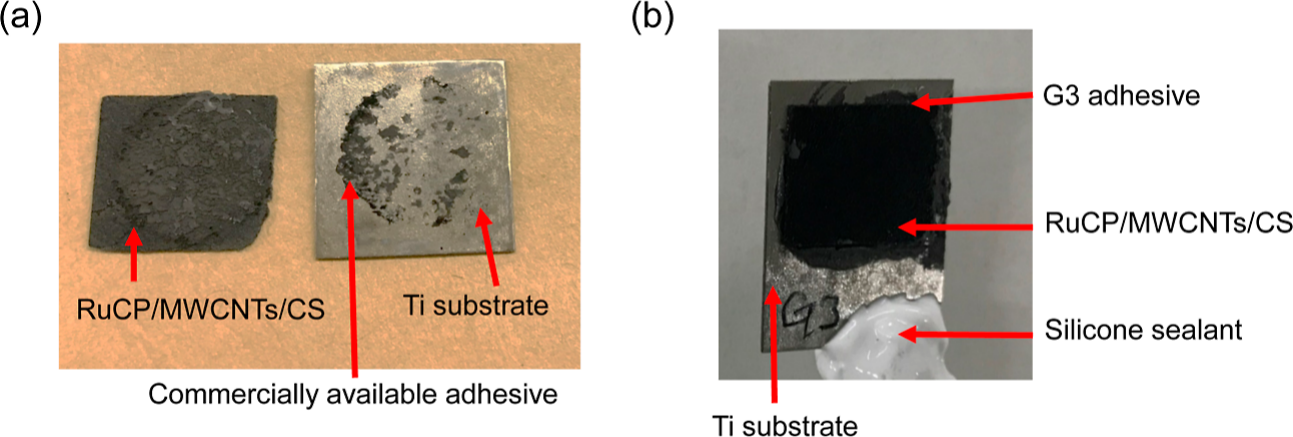
Appearance of 1 cm^2^ size cathodes composed of RuCP/MWCNTs/CSs and Ti plates (a) bonded using the commercially available adhesive observed after 110 h of operation and (b) bonded using the novel G3 graphite adhesive observed after 1000 h of operation.

We developed novel graphite adhesives to replace the commercially available adhesive previously used and prevent the peeling of RuCP/MWCNTs/CSs from the Ti plates. We conducted durability tests of three novel graphite adhesives (G1, G2, and G3; Table S1) as well as the previous adhesive. The RuCP/MWCNT/CS bonded to the Ti plate using the previous adhesive was easily peeled off when slightly touched after 110 h of operation, as shown in Figure 1a. In contrast, all the new adhesives exhibited sufficiently high adhesion. Table S1 summarizes the current densities of the cathodes using these adhesives. Among the three, G3 achieved the highest initial current density owing to its largest graphite composition and the resultant highest electric conductivity. On the other hand, there was no appreciable difference in the current density retention ratios, suggesting that the G3 adhesive guaranteed sufficiently high adhesion despite the smallest PVDF composition. The RuCP/MWCNT/CS was tightly bonded to the Ti plate using G3 even after a longer operation time of 1000 h, as displayed in Figure 1b.

However, the heat treatment at 100 °C for 3 h required for secure bonding using G3 was detrimental to the organic RuCP catalyst. Figure S1 compares the pre- and postloading processes. The current density for the preloading decreased more rapidly than that for the postloading, although it was larger than that for the previous adhesive. This suggests that RuCP was degraded by the heat treatment. Consequently, the G3 adhesive with the postloading process was adopted to prepare the newly developed EC reactors referred to as Type-A hereinafter.

**Figure 2:**
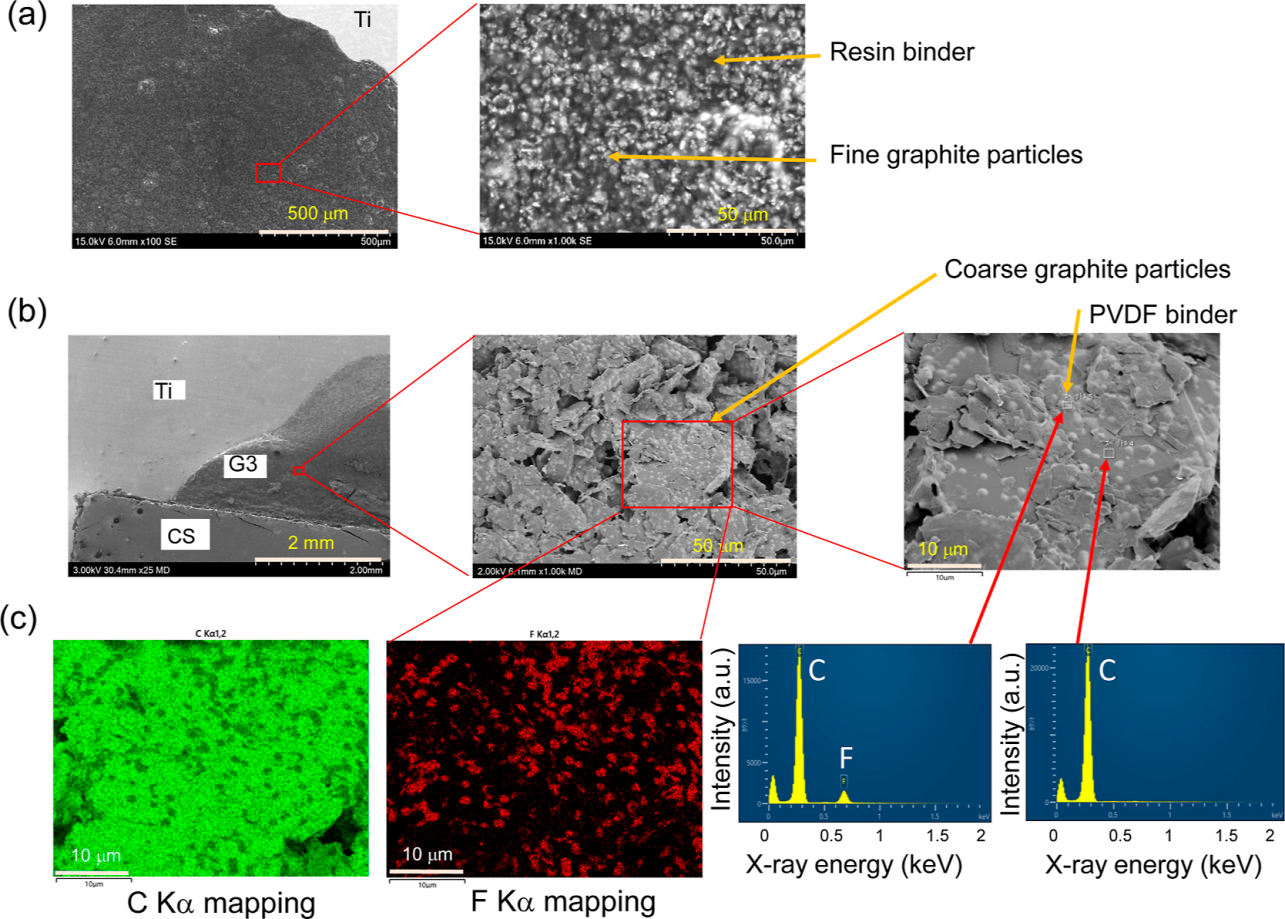
Surface SEM images of the (a) commercially available graphite adhesive and (b) novel G3 graphite adhesive. (c) EDX elemental mapping of G3 in the same area as that of the right photograph in (b) and EDX spectra at two given analysis sites.

Figure 2a,b displays the surface scanning electron microscopy (SEM) images of the two kinds of carbon adhesives. The commercially available adhesive consists of fine graphite particles dispersed in a resin binder. As the weight ratio of the binder was as high as 90 wt %, in addition to the small size of the graphite particles, the origin of the weakened adhesion and the resultant peeling off can be attributed to the swelling of the binder in the electrolyte rather than the cracking starting from the binder–graphite interfaces. In contrast, in the G3 adhesive, the weight ratio of the PVDF binder was only 13%. Therefore, the surfaces of the coarse graphite particles of several tens of μm in size were partially covered with the PVDF binder grains smaller than 1 μm, as is clear from the results of elemental analyses by energy-dispersive X-ray spectroscopy (EDX) depicted in Figure 2c. Nevertheless, the strong chemical resistance of PVDF guaranteed high adhesion.

### Strong Immobilization of the RuCP and Improvements in the Durability by Introducing an Amide Linkage between the RuCP and Carbon Supports

**Figure 3:**
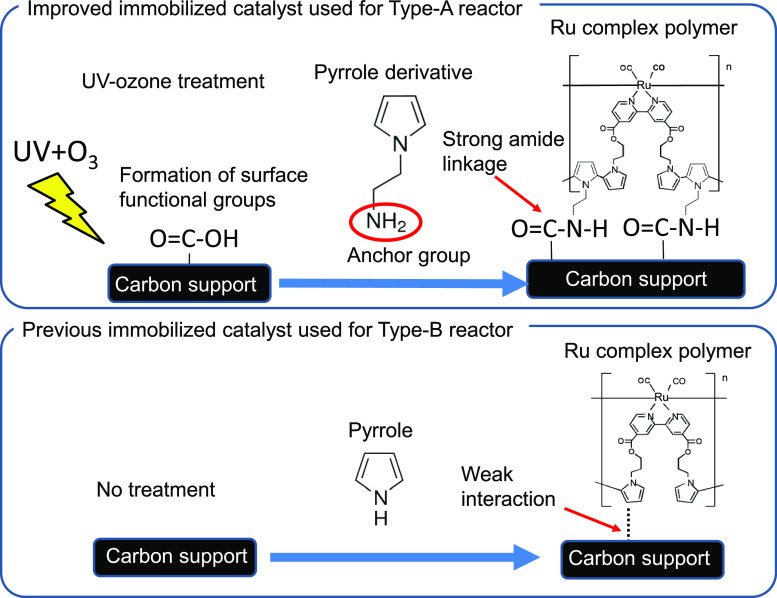
Presumed immobilization mechanism of the RuCP loaded into the UV–ozone-treated carbon support via the strong amide linkage between them.

The Ru complex and pyrrole were copolymerized with the help of the FeCl_3_ catalyst in the solution because the Ru complex is also equipped with a pyrrole group and loaded into the porous carbon supports of the MWCNT/CSs (pore diameter of 30 μm on average) by dropping the RuCP solution. Owing to the high electric conductivity of the pyrrole-based RuCP, electrons are supplied from the carbon supports to the active sites on the Ru complex, leading to formate production. However, the RuCP detached during the operation because the bonding force between the RuCP and the carbon supports was weak. To strengthen the bonding force, we adopted a UV–ozone treatment and pyrrole derivatives with an ethylamino group. The UV–ozone treatment on carbon materials has been reported to create oxygen-containing functional groups including a carboxyl group (−COOH) on the carbon surfaces.^26−28^ On the other hand, the carboxyl group interacts with the amino group, which functions as an anchor group to form a strong chemical bond through an amide linkage.^29^ Thus, the RuCP composed of pyrrole derivatives equipped with amino groups should be strongly immobilized to the carbon supports. This immobilization mechanism is illustrated in Figure 3a in comparison with the weak interaction between the previous RuCP and carbon supports depicted in Figure 3b.

Another means for strong immobilization is an increase in the amount of pyrrole derivative in the RuCP. In the previously used RuCP, the molar ratio of pyrrole to the Ru-complex monomer was as low as 0.004. Thus, greater amounts of pyrrole derivative up to 2 mol equiv to the Ru-complex monomer were introduced to increase the number of strong amide linkages. The compositions of the prepared RuCP solutions are summarized in Table S2.

To evaluate the effects of pyrrole derivative equipped with an amino group, we first prepared two types of cathodes using the same materials and processes, except for the use of different RuCP solutions of Sol. 0 (pyrrole) and Sol. 1 (pyrrole derivative). Sol. 1 contained the same amount of pyrrole derivative as that of pyrrole in Sol. 0. Figure S2a shows that the current densities of the two cathodes were almost the same in the initial stage of *I*–*t* measurements. Both formate FEs were close to unity, as depicted in Figure S2b. This indicates that the amide linkage did not have an adverse effect from the perspective of electron transfer from the carbon support to the RuCP. After a rapid decrease during the first 100 h, the difference in the current densities between these two cathodes gradually widened, although these two values decreased moderately; the pyrrole derivative exhibited larger values. The formate FEs changed in a similar manner. Thus, we confirmed that the introduction of the pyrrole derivative equipped with an amino group coupled with the UV–ozone treatment improved the durability.

Next, we examined the effect of amount of pyrrole derivative. The results of the durability test are summarized in Table S2 and Figure S3. Although 100 times more amount of the pyrrole derivative was introduced (Sol. 2), no appreciable differences were observed in either the current density or formate FE compared to the original amount (Sol. 1). In contrast, when the amount of pyrrole derivative was further increased to 1 mol equivalent to the Ru monomer (Sol. 3), the decrease in the current density and formate FE during the operation was notably reduced, along with a slight increase in the initial current density. In short, both higher initial performance and higher durability were achieved. In particular, formate FE was close to unity even after 600 h of operation. This is a significant improvement in contrast to formate FEs for Sol. 1 and Sol. 2 that are lowered to around 90%. The effects on the initial performance and durability were further enhanced by increasing the amount of FeCl_3_ (Sol. 4) and the pyrrole derivative (Sol. 5). Thus, we realized a higher durability of the RuCP with a higher catalytic activity by introducing more pyrrole derivatives, resulting in a greater number of strong amide linkages between the RuCP and carbon supports. Formation of the amide linkage was confirmed by FT-IR measurements on the pyrrole derivative loaded into the UV–ozone-treated carbon supports. Four peaks attributed to the secondary amide group were observed in the spectra displayed in Figure S4.^30^

Thus, we succeeded in developing highly durable cathodes by introducing a suitable amount of pyrrole derivative equipped with an amino group (Sol. 5), in addition to the newly developed novel graphite adhesive (G3). Consequently, the RuCP solution of Sol. 5 was adopted along with the UV–ozone treatment to prepare the newly developed Type-A EC reactors, as well as the G3 adhesive and postloading process.

### Highly Durable 1 cm^2^ Sized EC Reactors

In addition to the development of highly durable cathodes described in the previous subsections, we examined the durability of commercially available anodes. Three means were adopted in the new anodes: high-temperature firing,^31^ introduction of Pt–metal oxide interlayers,^32^ and mixing TaO_*x*_ with IrO_*x*_.^33,34^ We compared the electrochemical properties of the new anode with those of the previously used IrO_*x*_ particle/Ti anode. The results are presented in Figure S5, showing extremely more stable operation up to 1000 h of the new anode, although the initial activity was slightly lower. Thus, highly durable IrO_*x*_-TaO_*y*_/Pt-metal oxide/Ti anodes were employed for Type-A EC reactors.

**Figure 4:**
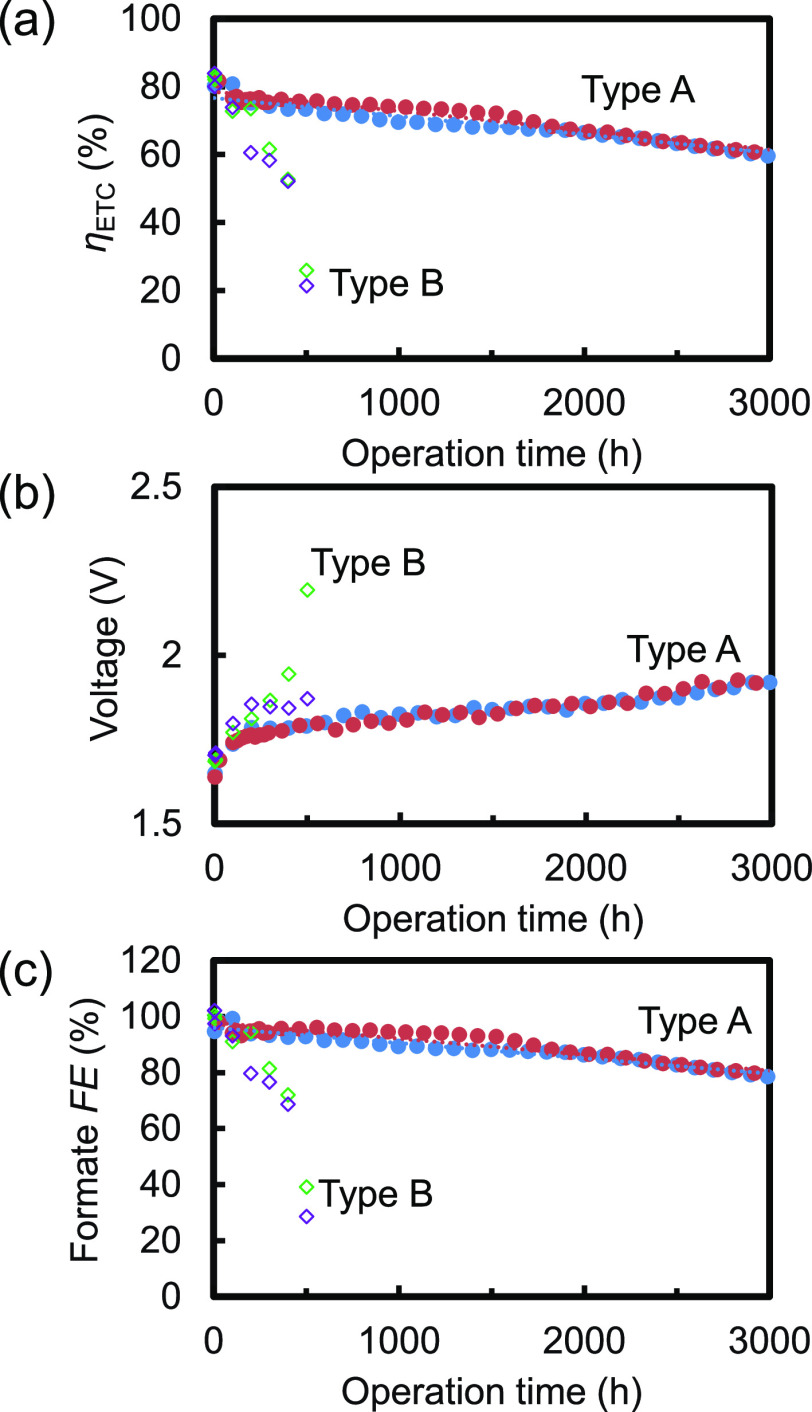
(a) Electric-to-chemical energy conversion efficiency (*η*_ETC_), (b) voltage, (c) formate FE of the 1 cm^2^ size Type-A and Type-B EC reactors during the long-term continuously operating durability tests. Type-A EC reactors adopted the IrO_*x*_-TaO_*y*_/Pt-metal oxide/Ti anodes and RuCP/MWCNTs/CS/G3 adhesive/Ti cathodes with the UV–ozone treatment, Sol. 5 including the large amount of pyrrole derivative equipped with an amino group, and postloading, while Type-B adopted the IrO_*x*_/Ti anode and cathodes using the commercially available adhesive, Sol. 0 including pyrrole, and preloading. Two EC reactors were tested for Type A (filled circles) and Type B (open diamonds).

The highly durable IrO_*x*_-TaO_*y*_/Pt-metal oxide/Ti anodes were adopted to construct 1 cm^2^ size Type-A EC reactors with the newly developed RuCP/MWCNTs/CS/G3 adhesive/Ti cathodes, in which UV–ozone treatment, Sol. 5 including a large amount of the pyrrole derivative equipped with amino groups, and postloading were incorporated. The results of long-term durability test of Type-A are compared in Figure 4 with those of the combination of the previously used anode and cathode referred to as Type-B hereinafter. At the beginning, *η*_ETC_ of 80% for Type-A was approximately the same as that for Type-B, as shown in Figure 4a, because the higher cathode activity and lower anode activity for Type-A than those for Type-B, respectively, canceled each other out. With increasing operation time, the voltage of Type-B increased, the formate FE lowered, as is clear from Figure 4b,c, respectively, and consequently *η*_ETC_ rapidly lowered. Eventually, *η*_ETC_ of Type-B was around 20% after 500 h of operation. The deterioration rate of *η*_ETC_ was 0.10%/h, determined from the linear fitting. In contrast, the performance of Type-A was stable. Although formate FE gradually decreased, a high value of 80% was achieved even after 3000 h of operation. The voltage increased, and therefore the *η*_ETC_ also lowered but slowly. As a result, the deterioration rate of *η*_ETC_ was as low as 0.0053%/h.

The amounts of Ru and Ir dissolved in the electrolytes during the durability tests were quantified, and they are summarized in Table 1. For the Type-B EC reactor, 55.2% of the total amount of RuCP was detached between 300 and 400 h, suggesting that the remaining RuCP was significantly smaller than the half after the 500 h operation. In contrast, the detachment rate of RuCP for Type-A was around 1%/100 h or lower. Thus, we prove the effect of strong immobilization of the RuCP owing to the strong amide linkage between the pyrrole derivative equipped with an amino group and UV–ozone-treated carbon supports. Furthermore, detachment or elution of Ir was not observed for the IrO_*x*_-TaO_*y*_/Pt-metal oxide/Ti anode used in Type-A, which also contributed to the high durability of the Type-A EC reactors. In contrast, an appreciable amount of Ir was dissolved in Type-B.

**Table 1:** Amounts of Ru and Ir Detached from the Catalyst Supports and Dissolved in the Electrolyte for the 1 cm^2^ Sized Type-A and Type-B EC Reactors during the Long-Term Continuously Operating Durability Tests, Measured by Inductively Coupled Plasma–Mass Spectrometry^a^

type	time of sampling	concentration of Ru (ng/mL)	detachment rate of Ru (%/100 h)	concentration of Ir (ng/mL)	detachment rate of Ir (%/100 h)
A (improved)	before	10		ND	
A (improved)	after 200 h	22	0.35	ND	ND
A (improved)	after 1100 h	85	1.34	ND	ND
A (improved)	after 2700 h	35	0.55	ND	ND
B (previous)	before	10		ND	
B (previous)	after 400 h	3500	55.2	8.9	1.2

a Type-A EC reactor adopted the IrO_*x*_-TaO_*y*_/Pt-metal oxide/Ti anode and RuCP/MWCNTs/CS/G3 adhesive/Ti cathode with the UV–ozone treatment, Sol. 5 including the large amount of pyrrole derivative equipped with an amino group, and post-loading, while Type-B adopted the IrO_*x*_/Ti anode and the cathode using the commercially available adhesive, Sol. 0 including pyrrole, and pre-loading. The electrolyte was replaced with a fresh one every 100 h. The Ir concentrations for Type-A were lower than the detection limit of 5 ng/mL, and hence the detachment rates were smaller than 0.66%/100 h. The concentrations of “before” were the values measured immediately after the electrodes were immersed in the electrolytes.

**Figure 5:**
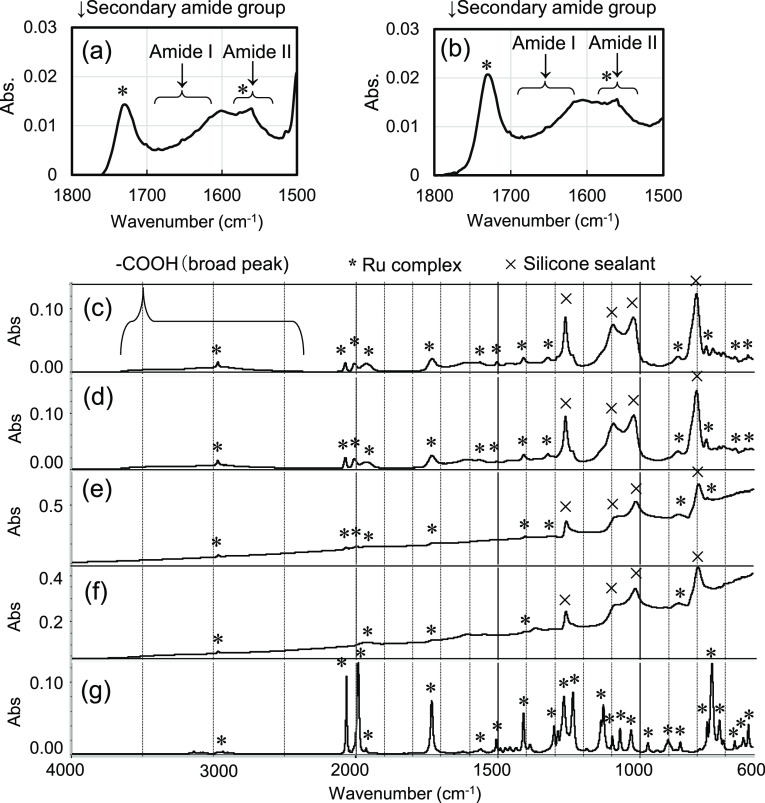
FT-IR spectra relevant to the amide linkage for the RuCP/MWCNTs/CS/G3 adhesive/Ti cathodes with the UV–ozone treatment and the pyrrole derivative equipped with an amino group (Type A) (a) before and (b) after the 3000 h durability test. FT-IR spectra relevant to the Ru complex for (c,d) Type-A before and after the durability test, respectively. (e,f) RuCP/MWCNTs/CS/previous adhesive/Ti cathodes without an amino group or the UV–ozone treatment (Type B) before and after the durability test, respectively, and (g) the Ru complex monomer. Note that the silicone sealant was present on the cathode surfaces.

Formation of the amide linkage between the RuCP and carbon supports in Type-A was confirmed by FT-IR measurements. Two absorption bands attributed to the secondary amide group labeled as amide I and amide II, respectively, were observed in the FT-IR spectra shown in Figure 5.^30^ Further, there were no significant changes in these absorption bands after the 3000 h durability test. This suggests notably high strength and durability of the amide linkage.

Another possible cause for the decrease in *η*_ETC_ is changes in the molecular structure of the Ru complex. Figure 5c,d compares the FT-IR spectra of Type-A cathodes before and after the 3000 h durability test. All of the peaks attributed to the Ru complex (see Figure 5g) remained unchanged after the durability test. This suggests high stability of the molecular structure of the Ru complex. On the other hand, the peak intensities of the Ru complex reduced, and the background in the low-wavenumber range increased in the spectra for Type-B displayed in Figure 5e,f, owing to the notable detachment of the RuCP.

### Proof of Improved Durability of the Inclined 75 cm^2^ Sized EC Reactor toward Practical Applications of Integrated EC–PV Cells

**Figure 6:**
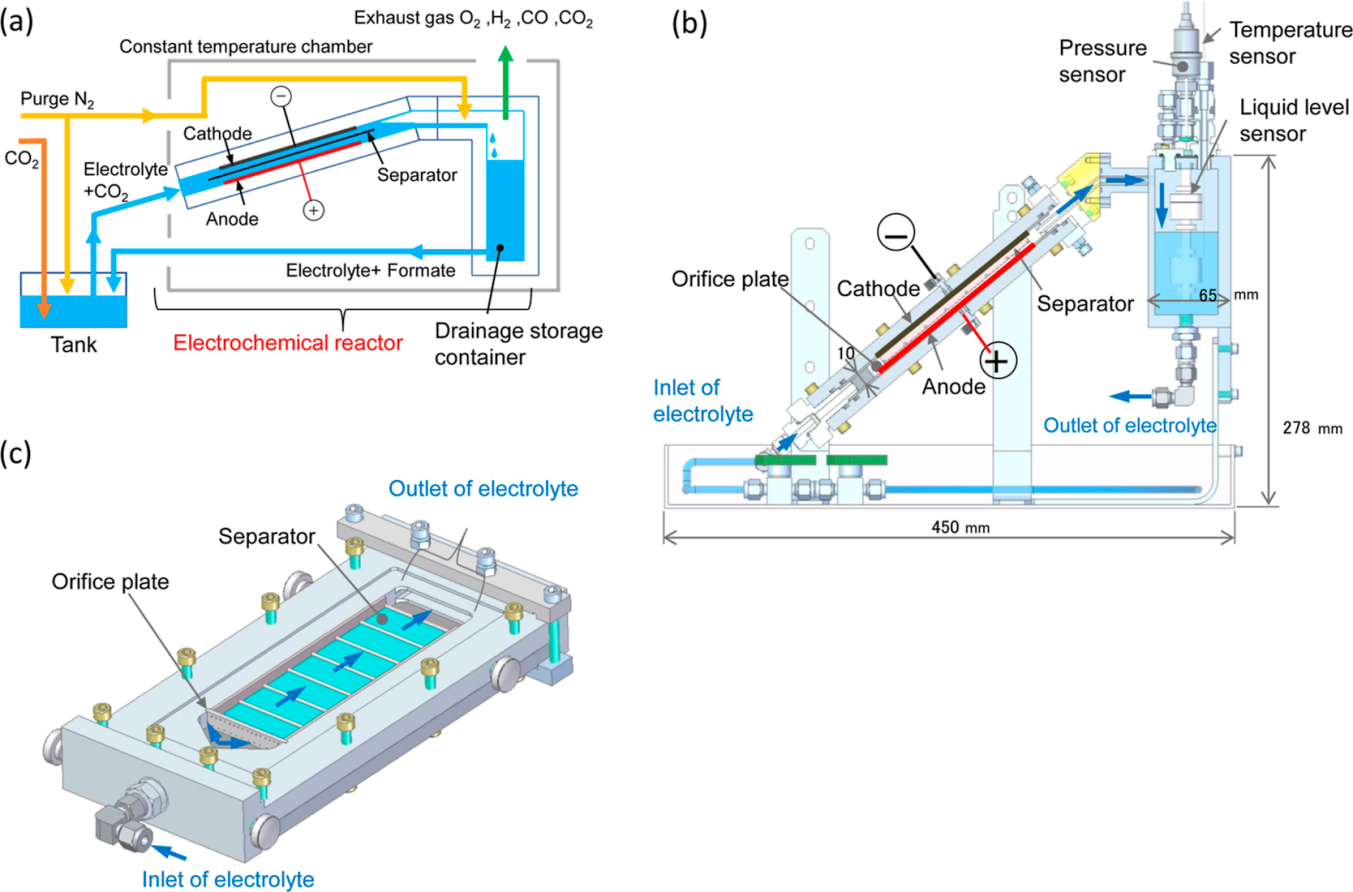
(a) 75 cm^2^ size Type-A EC reactor used for the EC–PV integrated artificial photosynthetic cell and the evaluation system. (b) Configuration diagram of the EC reactor viewed from the side. (c) Electrolyte flow channel in the EC reactor. 100% CO_2_ gas was bubbled into the 0.4 M KPi electrolyte in the tank prior to and during the evaluation. The CO_2_-saturated electrolyte was injected into the reactor with a flow rate of 30 mL/min. The electrolyte in the drainage storage container was pumped out when the liquid level exceeded a predetermined point. The electrolyte was returned to the electrolyte tank and thus circulated. The temperature of the electrolyte in the drainage storage container was 27–28 °C, approximately the same as the setting temperature of the constant temperature chamber. A pressure sensor was applied for emergency stop.

**Figure 7:**
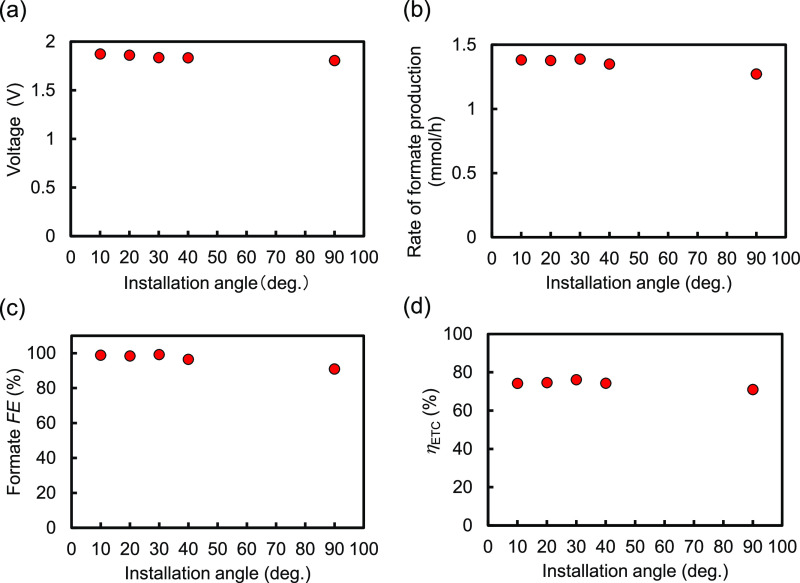
(a) Voltage, (b) rate of formate production, (c) formate FE, and (d) electric-to-chemical energy conversion efficiency (*η*_ETC_) of the 75 cm^2^ size Type-A EC reactors dependent on the installation angle, operating at a constant current of 75 mA for 17 h. These reactors adopted the IrO_*x*_-TaO_*y*_/Pt-metal oxide/Ti anodes and RuCP/MWCNTs/CS/G3 adhesive/Ti cathodes with the UV–ozone treatment, Sol. 5 including the large amount of pyrrole derivative equipped with an amino group, and postloading.

We scaled up the newly developed 1 cm^2^ sized EC reactors of Type-A to construct larger EC reactors of an electrolyte-flow type to prove their practical performance. Figure 6 schematically shows the configuration, while Figure S6 displays the appearance. An anode–cathode pair with an active area of 75 cm^2^ (5 × 15 cm) was set in the housing with the porous hydrophilic ultrahigh-molecular-weight polyethylene separator inserted between them. Figure 7 depicts the characteristics of formate production for the 75 cm^2^ size Type-A EC reactor dependent on the installation angle. Both the voltage and formate production rate shown in Figure 7a,b, respectively, were scarcely affected by the angle. These resulted in few impacts on formate FE and *η*_ETC_, as is clear from Figure 7c,d, respectively. Thus, detrimental impacts of inclined installation were scarcely observed, even though the anode and cathode faced each other in the single chamber. This is in contrast to a previous report in which the crossover reaction was notable in an inclined single-chamber EC reactor for water splitting.^23^ The porous separator inserted between the anode and the cathode prevented the O_2_ bubbles generated on the anode from transferring to the cathode. This is a significant advantage for practical applications because sunlight shines in inclined directions. In midlatitude areas, 30° is approximately the optimal installation angle for receiving more solar energy in the outdoors.^24,25^ Therefore, we conducted a long-term durability test on the EC reactor at an installation angle of 30°.

Another point for practical use is that EC reactors powered by solar cells operate intermittently only during the daytime in the outdoors. Therefore, the reactors were operated for 17 h, followed by a break for 7 h. The voltage increased gradually during the single-cycle operation, as shown in Figure S7. However, the voltage recovered to be close to that at the beginning of the previous cycle except for the first several cycles. A small portion of O_2_ bubbles generated on the anode was trapped and accumulated on the separator. This narrowed the effective separator area and enlarged the resistance for proton transfer from the anode to the cathode. However, the accumulated bubbles were removed during the nonoperation period, which is the mechanism underlying the voltage recovery. Thus, the intermittent operation lowered the voltage in average and consequently contributed to an improvement in η_ETC_.

**Figure 8:**
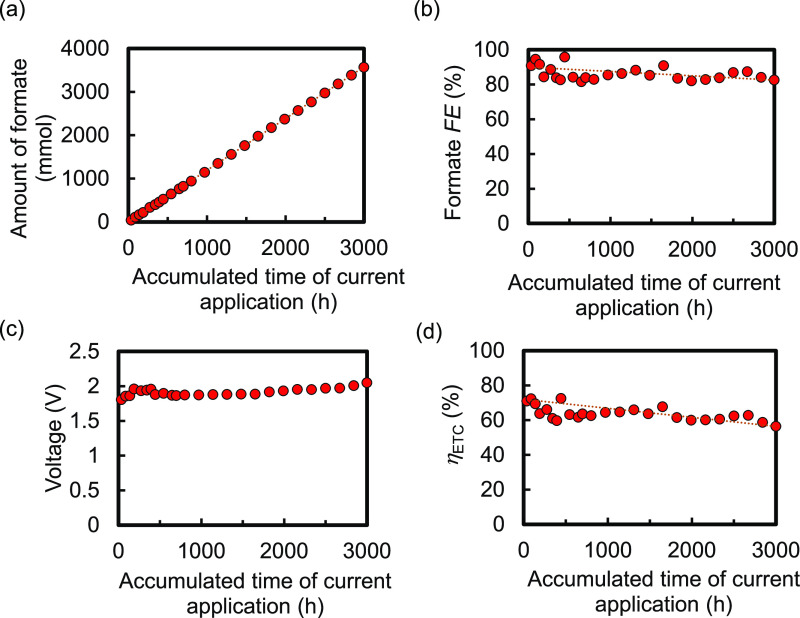
(a) Produced amount of formate, (b) formate FE, (c) voltage, and (d) electric-to-chemical energy conversion efficiency (*η*_ETC_) of the 75 cm^2^ size Type-A EC reactor installed at 30° during the long-term durability test with intermittent operation at a constant current of 75 mA for 17 h and at 0 mA for 7 h alternately in a day. The reactor adopted the IrO_*x*_-TaO_*y*_/Pt-metal oxide/Ti anodes and RuCP/MWCNTs/CS/G3 adhesive/Ti cathodes with the UV–ozone treatment, Sol. 5 including the large amount of pyrrole derivative equipped with an amino group, and postloading. A single Type-A EC reactor was tested.

Figure 8 shows the results of long-term durability test of the 75 cm^2^ size Type-A EC reactor installed at 30°. The horizontal axis indicates the cumulative operation time during intermittent operation. Figure 8a–c indicates that the amount of produced formate increased almost linearly over time up to 3000 h and a high FE over 80% was maintained, whereas the voltage gradually increased, respectively. As a result, *η*_ETC_ decreased moderately from 72% at the beginning to 58% after the 3000 h operation; around 80% of its initial value was maintained, as is clear from Figure 8d. Thus, the deterioration rate of *η*_ETC_ was 0.0053%/hour. This value was almost the same as that for the 1 cm^2^ size Type-A EC reactors shown in Figure 4, suggesting the high reliability of the resultant deterioration rate. As is clear from Table S3, it was confirmed that the detachment of the RuCP was also suppressed, as was the case with the 1 cm^2^ size Type-A EC reactors. Thus, we proved the high durability of the 75 cm^2^ size EC reactor under practical installation and operation conditions.

Table 2 summarizes the previously reported results of long-term durability tests on EC reactors for the CO2RR compared to the present results. The 3000 h operation with high durability realized in this study using the RuCP molecular catalyst was significantly longer than the other results of formate production using metal and carbon catalysts. Another advantage of the RuCP catalyst is its lower operating voltage compared to other catalysts. On the other hand, one of the shortcomings of the present EC reactors was the low current density of 1 mA/cm^2^. However, this limitation can be circumvented by stacking the anode and cathode electrodes. Indeed, eight-stacked anode and cathode electrodes powered by a PV module operated at a current density of 0.84 mA/cm^2^ in the EC–PV integrated cell of 1 m^2^ in size previously constructed for artificial photosynthesis, achieving a large current of 65 A at a low operating voltage of 1.7 V and consequently a high η_STC_ of 10.5%.^17^

**Table 2:** Benchmark of Durability of Various EC Reactors with Different Catalysts for the CO2RR^a^

EC reactor configuration (catalyst)	electrolyte (pH)	active area (cm^2^)	main product	operation time (h)	FE (%)	current density (mA/cm^2^)	operating voltage (V)	ref
single-chamber type and porous separator (RuCP with pyrrole derivative/MWCNTs/CS/G3 graphite/Ti)	0.4 M KPi (6.3)	75	formate	3000	95	1.0	1.8	this study
MEA (nanoparticle Sn on GDE)	DI water	5	formate	550	30	42	3.5	(35)
H-cell (Pd–Pb bimetallic on GDE)	0.5 M HCOOK + 0.1 M K_3_C_6_H_5_O_7_	1	formate	288	80	11	–1.8 (vs Ag/AgCl)	(36)
3-electrode cell (N-doped nanoporous carbon/CNT on carbon paper)	0.1 M KHCO_3_ (6.8)	1	formate	36	81	5.67	–0.8 (vs RHE)	(37)
3-electrode cell (boron-doped diamond electrodes)	0.075 M RbOH (6.2)	5	formate	24	10–60	20	–2.2 (vs Ag/AgCl)	(38)
MEA (Ag NPs on imidazolium-based polymer)	0.01 M KHCO_3_	5	CO	4380	95	50	3.0	(39)
MEA (Ag-based GDE)	0.4 M K_2_SO_4_/0.5 M KHCO_3_ (7)	10	CO	1200	70	300	7–7.5	(40)
3-electrode cell (triangular Ag NPs and carbon black on glassy carbon)	0.1 M KHCO_3_	0.785	CO	168	96	1.2	–0.86 (vs RHE)	(41)

a EC reactors other than that used in this study were installed vertically on the ground.

However, a longer operation time with high durability and a larger current density have been realized for CO production using an Ag nanoparticle catalyst with an electrolyte membrane composed of an imidazolium salt as an ionic liquid.^39^ Gas diffusion electrodes (GDEs) and membrane electrode assemblies are often used to achieve high current densities.^35,39,40^ However, Ag-based catalysts suffer from notably high operating voltages.

Thus, our approach using a strongly immobilized molecular catalyst realized both high durability and low operating voltage, although the current density was low. The use of GDE and a gas-phase CO_2_ supply can increase the current density.^42,43^

## Conclusions

We have drastically improved the long-term durability of the 75 cm^2^ size EC reactor designed for producing formate from CO_2_ and water in the integrated EC–PV cells. This reactor maintained a high FE of over 80% and a high *η*_ETC_ of around 60% after 3000 h of operation under practical conditions. We addressed the two major causes for the inadequate durability of the previously constructed 1000 cm^2^- and 1 m^2^ size reactors. The introduction of strong amide linkage between the RuCP cathode catalyst and carbon supports suppressed the detachment of the RuCP. The newly developed chemically resistant graphite adhesive prevented the carbon supports from peeling off from the Ti plates. In addition, the previously used IrO_*x*_/Ti anodes, which suffered from dissolution of Ir into the electrolyte, were replaced with highly durable IrO_*x*_-TaO_*y*_/Pt-metal oxide/Ti anodes. Furthermore, we investigated two potential issues for practical applications. Although inclined installation increased incident solar energy, it often promoted crossover reactions. This issue was solved by inserting a porous separator that impedes the transfer of the O_2_ bubble from the anode to the cathode. However, the use of a separator raised another issue: the number of trapped O_2_ bubbles on the separator narrowed the effective separator area. Intermittent operation corresponding to changes in solar energy during the day mitigated the detrimental impact of the second issue. Thus, we established the groundwork for the widespread use of integrated EC–PV cells for artificial photosynthesis, demonstrating high durability and exceptional FE and *η*_ETC_.

## Experimental Section

### Development of Highly Durable Cathode Electrodes

#### Preparation of Previously Used Cathodes

The cathodes previously adopted for the 1000 cm^2^ and 1 m^2^ sized EC reactors were prepared by using the following sequential processes: The Ru complex monomer [Ru{4,4′-di(1-*H*-1-pyrrolypropyl carbonate)-2,2′-bipyridine}(CO)_2_Cl_2_] was synthesized according to a previously established process.^14^ A 5 wt % multiwalled carbon nanotube (MWCNT) dispersion ink (Meijo Nano Carbon Co. Ltd.) and carbon sheets measuring 350 μm in thickness (CSs; TGP-H-120, Toray Industries, Inc.) were used as supports of the RuCP catalyst. The Ru complex monomer (5.02 × 10^–7^ mol/cm^2^) was dissolved in a mixture of ethanol (14 μL/cm^2^) and acetonitrile (53 μL/cm^2^), in the presence of pyrrole (1.99 × 10^–9^ mol/cm^2^) and iron chloride (FeCl_3_, 2.79 × 10^–6^ mol/cm^2^). Under these conditions, the Ru complex monomer and pyrrole were copolymerized. Then, the resultant RuCP was loaded into the MWCNTs/CS by dropping the RuCP solution into porous supports and then subjecting them to vacuum-drying, repeating the process 10 times.^17^ The surface of a 1.5 cm × 2 cm Ti plate (JIS Ti type 1; thickness: 0.5 mm) was mechanically polished. A 1 cm × 1 cm piece of the prepared RuCP/MWCNT/CS was bonded onto a mechanically polished Ti plate using a commercially available graphite adhesive (#15-1137, Okenshoji, distributed for bonding SEM specimens) and left to stand at room temperature overnight. The end of the Ti plate and a conducting wire with a terminal were mechanically connected. Finally, the terminal was covered with silicone rubber. These cathodes were also prepared in this study for clarifying the causes of degradation and compared with newly developed cathodes. Furthermore, they were used for constructing the previous type-B EC reactors with IrO_*x*_ anodes.

#### Chemically Resistant Novel Graphite Adhesives

The novel graphite adhesives were composed of KF polymer (L#1120, Kureha Corp.), which is a mixture of 12% polyvinylidene fluoride (PVDF) and 88% *N*-methylpyrrolidone (NMP), and graphite particles (KS44, Lonza) of 5–50 μm in size. As the graphite content increased, the electric conductivity increased, while the adhesion strength decreased. Therefore, we prepared adhesives with three different graphite/PVDF ratios (G1, G2, and G3; Table S1) to determine the optimal composition. The ingredients were mixed by using a revolutionary mixer. After applying 0.05 g/cm^2^ of the new graphite adhesive to the Ti plate, the MWCNT/CS was bonded, followed by heat treatment at 100 °C for 3 h.

The heat treatment required for the novel graphite adhesives degraded the catalytic activity of RuCP, as shown in Figure S1. Therefore, the newly developed cathode was prepared using the reverse procedure, that is, the carbon support was first bonded to the Ti plate using the novel graphite adhesive, and then the RuCP was loaded by the drop-drying process. This postloading process eliminated the detrimental impact of heat treatment, in contrast to the conventional preloading process.

Durability tests of these cathodes were conducted by using 1 cm^2^ size samples. A three-electrode configuration was adopted using the cathode as the working electrode with a platinum wire counter electrode and a Hg/Hg_2_SO_4_ reference electrode. 100% CO_2_ gas was bubbled through 80 mL of 0.4 M potassium phosphate buffer electrolyte (0.2 M K_2_HPO_4_ + 0.2 M KH_2_PO_4_, KPi) prior to and during the measurements. Current–time (*I*–*t*) measurements at a constant potential of −1.2 V vs Hg/Hg_2_SO_4_ were performed using a potentio-galvanostat (VMP3, Bio-Logic Sciences Instruments). Thus, the composition of the novel graphite adhesive was optimized.

#### Introduction of the Amide Linkage between the RuCP and Carbon Supports

The MWCNT/CSs bonded to the Ti plates using the newly developed novel graphite adhesive (G3) were subjected to UV–ozone treatment for 30 min by using a UV ozone cleaner (NL-UV253, Nippon Laser). RuCP solutions containing the pyrrole derivative 2-(1*H*-pyrrol-1-yl) ethanamine of five different amounts (Sol. 1–Sol. 5) listed in Table S2 were prepared using a procedure similar to that used for the previous RuCP solution. The solutions were then dropped onto the UV–ozone-treated MWCNT/CS/Ti-plates. The effect of the amount of FeCl_3_ was also examined along with that of the pyrrole derivative.

Durability tests for these cathodes were conducted in a manner similar to those of novel graphite adhesives. The *I*–*t* measurements at a constant potential of −1.2 V vs Hg/Hg_2_SO_4_ were periodically stopped, and the electrolyte was sampled to quantify the formate produced. The concentration of formate in the electrolyte was measured using an ion chromatograph (Integrion RFIC EG, Dionex Corp.) and converted to the formate FE. The accuracy of the measured data was ±5%. Thus, the composition of the RuCP solution was optimized.

The reduction reaction of CO_2_ to produce formate is described as follows
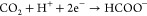
1the FE of formate production was calculated using the following formula
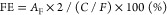
2where *A*_F_ is the amount of formate (mol), *C* is the charge (C), and *F* is the Faraday constant (96,485.3365 C/mol).

Prior to the long-term durability tests, we evaluated the effect of formate concentration dissolved in the electrolyte on the formate production performance. The results of current density and formate FE are shown in Figure S8a,b, respectively. The current density remained constant up to 15 mM, followed by a gradual decrease with increasing concentration. Although the formate FE was as high as 95% in the low formate concentration range, it started to decrease at 15 mM. Therefore, the electrolyte was periodically replaced with a fresh one before the formate concentration reached 20 mM to eliminate substantial decreases in the current density and formate FE.

### Alternative Anode Electrodes for High Durability

The anodes employed in previous Type-B EC reactors were prepared by loading IrO_*x*_ catalyst particles onto Ti plates. An IrO_*x*_ nanocolloid solution was dropped onto the Ti plates, followed by drying. The Ir content was approximately 60 μg/cm^2^. The detailed procedure has been reported elsewhere.^16,17,44^ Then, the IrO_*x*_ particles were immobilized by a vacuum-drying treatment at 60 °C for improving the durability of water oxidation.^22^

The previous anodes were replaced with highly durable anodes composed of IrO_*x*_-TaO_*y*_/Pt-metal oxide/Ti-plate commercially available (Mode-211H, Ishifuku Metal Industry) in Type-A reactors. The thickness of the IrO_*x*_-TaO_*y*_ catalyst layer was 1 μm. The detailed structures and fabrication processes are reported elsewhere.^32^ The durability tests of these anodes were conducted in a manner similar to those of the cathodes, except for the use of a Ag/AgCl reference electrode and the voltage–time (*V*–*t*) mode at a constant current of 1 mA.

### Long-Term Durability Test on 1 cm^2^ Sized EC Reactors

We prepared EC reactors with an active area of 1 cm^2^ using the new IrO_*x*_-TaO_*y*_/Pt-metal oxide/Ti anodes and newly developed cathodes with optimized graphite adhesion (G3), UV–ozone treatment, RuCP solution (Sol. 5), and postloading. These EC reactors are termed Type-A. For comparison, Type-B EC reactors consisting of previous cathodes and IrO_*x*_/Ti anodes were also prepared.

The EC reactor was placed in a 100 mL container with 80 mL of 0.4 M KPi electrolyte. *V*–*t* measurements were conducted at a constant current of 1 mA. 100% CO_2_ gas was bubbled through prior to and during the measurements. The operation was periodically stopped, the electrolyte was sampled, and the amount of produced formate was quantified using ion chromatography to determine the formate FE.

The amount of formate was converted to the energy conversion efficiency from the electric energy to the chemical energy (*η*_ETC_) according to the following formula
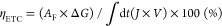
3where Δ*G* is the change in the Gibbs free energy per mole of formate produced from CO_2_ and water (Δ*G* = 270 kJ/mol at 298 K); *J*, *V*, and *t* are the current, voltage, and operation time, respectively.

### Construction of 75 cm^2^ Sized EC Reactors with Electrolyte Circulators toward Practical Application of Integrated EC–PV Cells

We constructed the large EC reactor of Type-A shown in Figure 6, whose appearance is displayed in Figure S6. The anode with an active area of 75 cm^2^ (5 × 15 cm) was located on the lower side when the EC reactor was installed at an inclined angle. This was so that the O_2_ bubbles generated on the anode did not cover the anode surface and they immediately flowed out to the exit. The CO_2_-dissolved KPi electrolyte in the tank (10 L) was injected from the bottom of the housing of the reactor at a flow rate of 30 mL/min and transferred between the anode and the cathode. The electrolyte including the produced formate was ejected from the top end and stored in the drainage storage container. Finally, the electrolyte was returned to the electrolyte tank and thus the electrolyte was circulated.

We addressed two challenges unique to scale-up. The first was the suppression of the crossover reaction, particularly the ORR, on the cathode. To solve this issue, a nanoporous film of hydrophilic ultrahigh-molecular-weight polyethylene (Miraim, Teijin Ltd.) was inserted as the separator between the anode and the cathode. The second challenge was ensuring a uniform flow of the electrolyte to supply sufficient CO_2_ to the cathode surface. For this purpose, an orifice plate was employed in the flow channel and designed using SOLID WORKS Flow Simulation software (Dassault Systèmes); see Table S4 and Figures S9 and S10.

The EC reactors were installed at angles of 10, 20, 30, 40, and 90° (vertical) to the ground in a constant-temperature chamber maintained at 28 °C. Then, they were operated at a constant current of 75 mA using a programmable power supply (P4K6–4-Lde, Matsusada Precision, Inc.). The voltage was monitored during the operation, and the electrolyte was periodically replaced with a fresh one. The formate concentration in the electrolyte was measured at regular intervals using ion chromatography and converted to formate FE.

### Characterization of Materials and Deterioration Analyses of the EC Reactors

SEM–EDX (SU7000, Hitachi High-Tech Co.) was used to observe the microscopic structures of graphite adhesives.

FT-IR measurements were adopted to investigate the molecular structures and bonding in the cathodes by using an FT-IR spectrometer (Nicolet iS50, Thermo Fisher Scientific) equipped with a deuterated triglycine sulfate (DTGS) detector and a single-reflection internal reflection element (IRE) of Ge. All the spectra were collected at a 4 cm^–1^ resolution and 64 scans. The background spectrum from IRE was acquired without the sample. For confirmation of the formation of the amide linkage between the pyrrole derivative and carbon supports, two model samples were prepared: the pyrrole derivative equipped with an amino group loaded into the UV–ozone-treated carbon support and pyrrole loaded into the carbon support without the UV–ozone treatment.

To evaluate the detachment of the RuCP cathode and IrO_*x*_-based anode catalysts, the amounts of Ru and Ir dissolved in the electrolytes were quantified by ICP–MS (8900, Agilent Technology) before and during the durability tests. The electrolyte samples of 0.4 M KPi including the dissolved Ru or Ir were diluted 1000 times for the ICP–MS measurements to lower the total ion concentration below the upper limit for quantification. FT-IR measurements of RuCP/MWCNTs/CS were applied to detect changes in the molecular structures of the Ru complex before and after the durability test.

## Supporting Information Available

Composition of new graphite adhesive, effect of heat treatment of new graphite adhesive on CO2RR, comparison of Ru complex polymer solution using pyrrole and pyrrole derivative, effect of pyrrole derivative on CO2RR, FT-IR analysis of amide linkage between the carbon support and pyrrole derivative, durability of the new IrO_*x*_-TaO_*y*_/Pt-metal oxide/Ti anode, appearance of the 75 cm^2^ size EC reactor and its operating characteristics, catalyst detachment analysis, and orifice patterns designed for the 75 cm^2^ size EC reactors and electrolyte flow simulation (PDF)
