# Supplementary material for: Enhancing Long-Term Durability of Electrochemical Reactors Producing Formate from CO2 and Water Designed for Integration with Solar Cells
Source: ACS Omega. 2024 Mar 1;9(10):11646–57. doi: 10.1021/acsomega.3c08911 (PMC10938335; doi:10.1021/acsomega.3c08911)
Supplement: Supplementary file 1 — ao3c08911_si_001.pdf [file ao3c08911_si_001.pdf]

Supporting Information

Enhancing long-term durability of electrochemical reactors producing formate from CO_2 and water designed for integration with solar cells

Naohiko Kato,[†,] Yasuaki Kawai,[†] Natsumi Nojiri,[†] Masahito Shiozawa,[†] Yoshihiro Kikuzawa,[†] Nobuaki Suzuki,[†] Satoru Kosaka,[†] Yuichi Kato,[†] Juntaro Seki,[†] Tsuyoshi Hamaguchi,[†] and Yasuhiko Takeda[†]*

[†]Toyota Central R&D Labs., Inc. Nagakute, Aichi, 480-1192 Japan

Corresponding Author

*Naohiko Kato. **n-kato@mosk.tytlabs.co.jp** **Phone. No. +81-561-71-7989**

ORCID: https://orcid.org/0000-0001-9158-1549

Number of pages: 15
Number of tables: 4
Number of figures:10

Table S1. Comparison among the newly developed novel graphite adhesives and the commercial one. The 1 cm^2-sized cathodes were prepared using the same materials and processes as those of the Type-B cathodes, except for the use of different adhesives. Current–time (*I*–*t*) measurements were conducted at a constant potential of −1.2 V vs. Hg/Hg_2SO_4. G3 achieved the highest initial current density, while all of G1−G3 guaranteed sufficiently high chemical resistance. Consequently, the G3 adhesive was adopted for preparing the newly developed Type-A EC reactors.

**Code**	**G1**	**G2**	**G3**	**Commercially available**
Weight ratio of KF polymer L#1120	1	1	1	–
Weight ratio of graphite KS44	0.25	0.5	0.75	–
Ratio of weight of graphite after cure (%)	68	80	87	<10
Initial current density (mA/cm^2)	2.9	4.0	4.2	3.5
Current-density retention ratio after 100 h operation (%)	59	53	58	50
Adhesion between Ti and RuCP/MWCNTs/CS after 110−1000 h operation	Good (not peeled) after 1000 h	Good (not peeled) after 1000 h	Good (not peeled) after 1000 h	No good (peeled) after 110 h

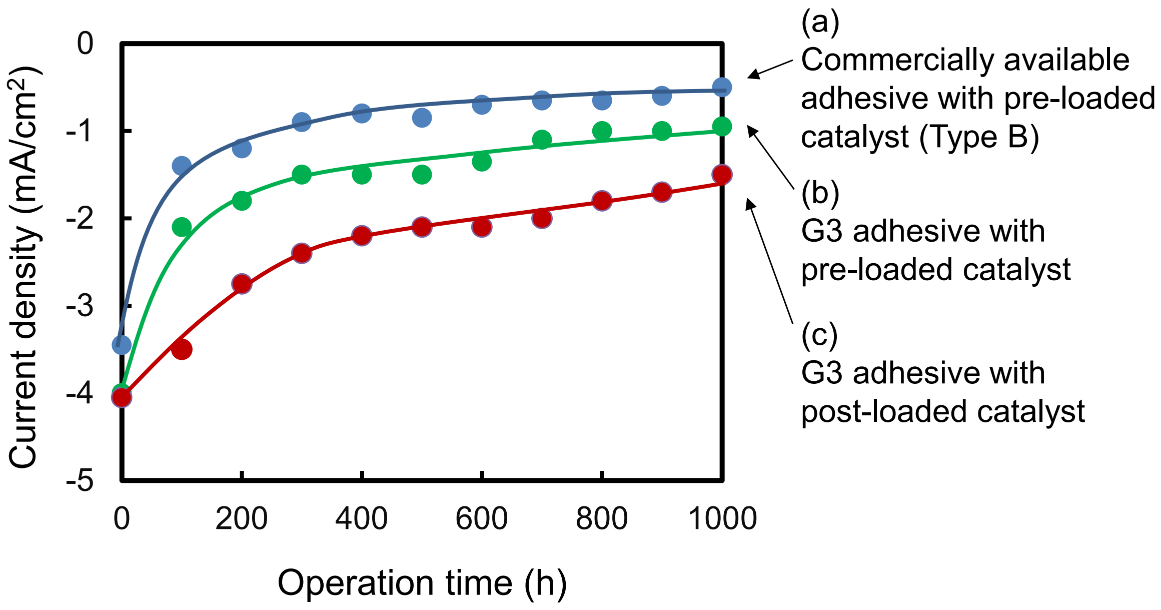

Figure S1. Impact of the heat treatment at 100 °C associated with the bonding using the novel G3 adhesive on the CO2RR performance. The 1 cm^2-sized cathodes were prepared using the same materials and processes as those of the Type-B cathodes, except for the use of different graphite adhesives and RuCP loading processes. (a) Type-B, (b) The RuCP catalyst was pre-loaded onto the MWCNTs/CS before bonded on the Ti plate using the G3 adhesive, and (c) The RuCP catalyst was post-loaded onto the MWCNTs/CS after bonded on the Ti plate using the G3 adhesive. Current–time (*I*–*t*) measurements were conducted at a constant potential of −1.2 V vs. Hg/Hg_2SO_4. The heat treatment was found to degrade the CO2RR performance of the RuCP catalyst from the comparison between (b) and (c), although (b) exhibited higher performance than (a). Consequently, the G3 adhesive with the post-loading process was adopted for preparing the newly developed Type-A EC reactors.

Table S2. Comparison among the RuCP solutions using pyrrole or the pyrrole derivative with different amounts. The 1 cm^2-sized cathodes were prepared using the same materials and processes as those of the Type-A cathodes, except for the use of Sol. 0 or different RuCP solutions composed of the pyrrole derivative (Sol. 1–Sol. 5). A mixed solvent composed of 53 $\mu L/cm^2$ of acetonitrile and 14 $\mu L/cm^2$ of ethanol was used. Current–time (*I*–*t*) measurements were conducted at a constant potential of −1.2 V vs. Hg/Hg_2SO_4. Sol. 5 including the largest amount of the pyrrole derivative and $FeCl_3$ achieved the highest values of the current density times formate *FE* both in the initial and after 600 h measurements. Consequently, Sol. 5 was adopted for preparing the newly developed Type-A EC reactors. See also Figures S2 and S3.

Type	Sol. 0 (Previous)	Sol. 1	Sol. 2	Sol. 3	Sol. 4	Sol. 5
Ru complex (mol/cm^2)	5.02×10^{-7}	5.02×10^{-7}	5.02×10^{-7}	5.02×10^{-7}	5.02×10^{-7}	5.02×10^{-7}
Pyrrole (mol/cm^2)	1.99×10^{-9} (1 time)	–	–	–	–	–
Pyrrole derivative (mol/cm^2)	–	1.99×10^{-9} (1 time)	1.99×10^{-7} (100 times)	5.02×10^{-7} (252 times)	5.02×10^{-7} (252 times)	1.00×10^{-6} (504 times)
$FeCl_3$ (mol/cm^2)	2.79×10^{-6} (1 time)	2.79×10^{-6} (1 time)	2.79×10^{-6} (1 time)	2.79×10^{-6} (1 time)	4.19×10^{-6} (1.5 times)	5.57×10^{-6} (2 times)
Initial current density, J (mA/cm^2)	2.5	2.5	2.3	2.7	3.4	4.4
Initial formate *FE* (%)	90.7	91.5	87.1	100.6	95.1	94.7
Initial $J \times FE$	226.8	232.3	197.0	262.7	324.6	412.0
J after 600 h (mA/cm2)	0.69	1.0	0.8	1.2	1.1	1.3
FE after 600 h (%)	79	87.1	89.9	96.5	99.1	99.1
$J \times FE$ after 600 h	54.5	84.1	71.1	119.3	109.8	127.5

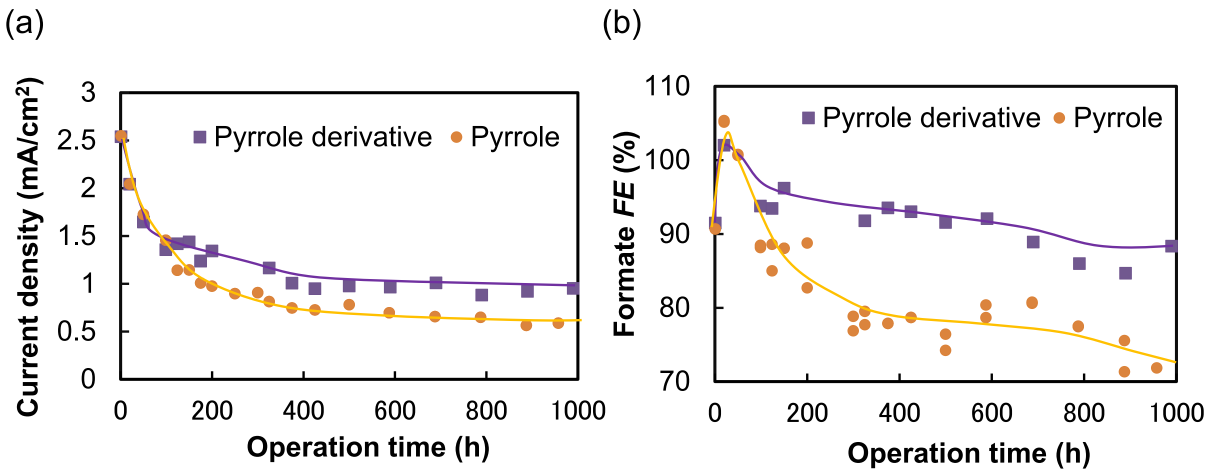

Figure S2. Effects of the pyrrole derivative on the CO2RR performance. The 1 cm^2-sized cathodes were prepared using the same materials and processes as those of the Type-A cathodes, except for the use of Sol. 0 (pyrrole) and Sol. 1 (pyrrole derivative); see Table S2. Current–time (I–t) measurements were conducted at a constant potential of −1.2 V vs. Hg/Hg_2SO_4. The pyrrole derivative improved the durability with the same initial performance as that for the pyrrole.

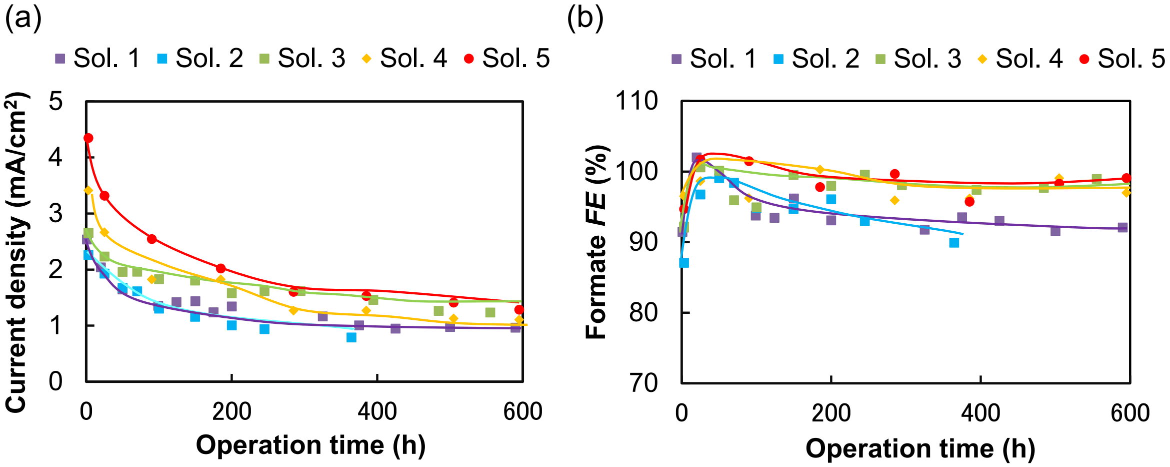

Figure S3. Effects of the pyrrole derivative on the CO2RR performance. The 1 cm^2-sized cathodes were prepared using the same materials and processes as those of the Type-A cathodes, except for the use of the RuCP solutions composed of different amounts of the pyrrole derivative; see Table S2. Current–time (I–t) measurements were conducted at a constant potential of −1.2 V vs. Hg/Hg_2SO_4. Sol. 5 including the largest amount of the pyrrole derivative and $FeCl_3$ achieved the highest current density and formate *FE* among the five RuCP solutions. Consequently, Sol. 5 was adopted for preparing the newly developed Type-A EC reactors. See also Table S2.

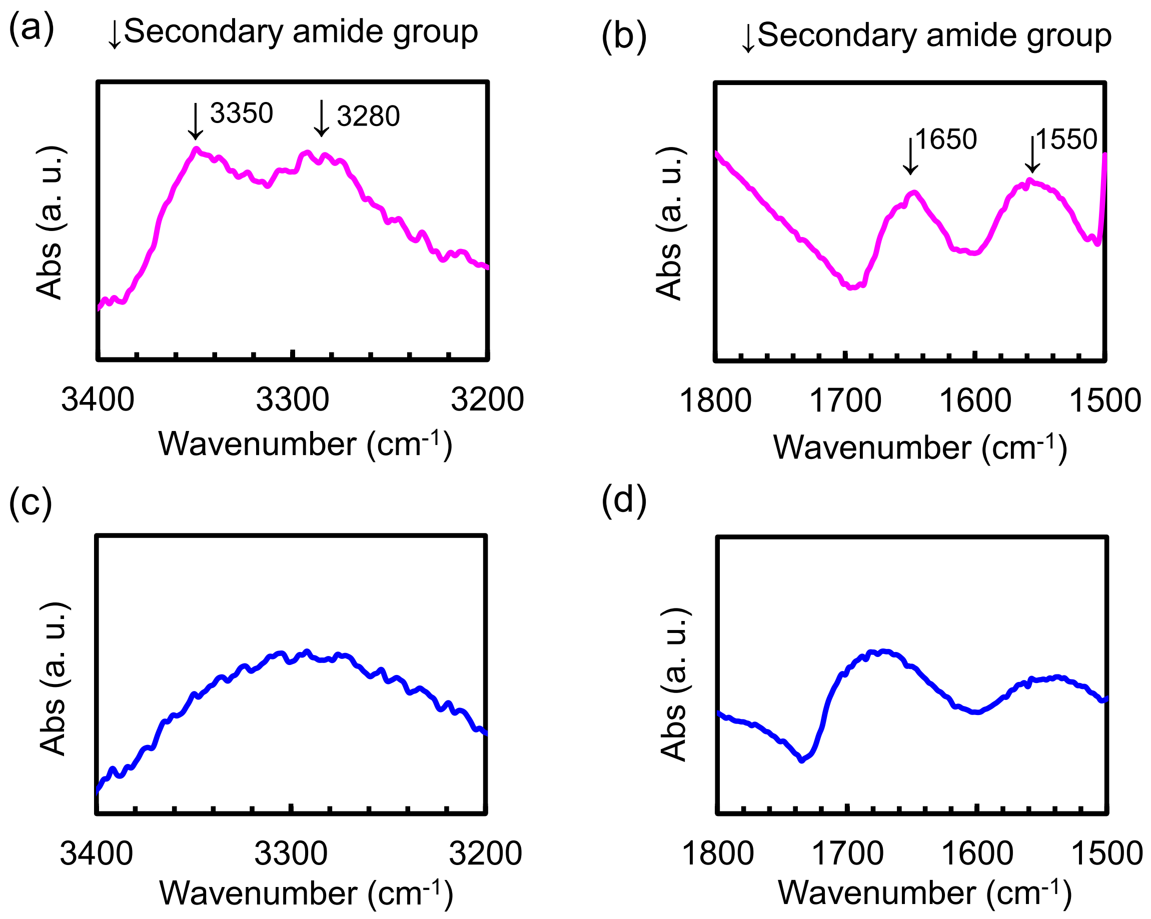

Figure S4. FT-IR spectra relevant to the amide linkage, (a) and (b) for the pyrrole derivative equipped with amino group loaded onto the UV-ozone-treated carbon support; and (c) and (d) for pyrrole loaded onto the carbon support without the UV-ozone treatment. In the spectra of (a) and (b), peaks characteristic to secondary amide groups originating from the amide linkage are exhibited.

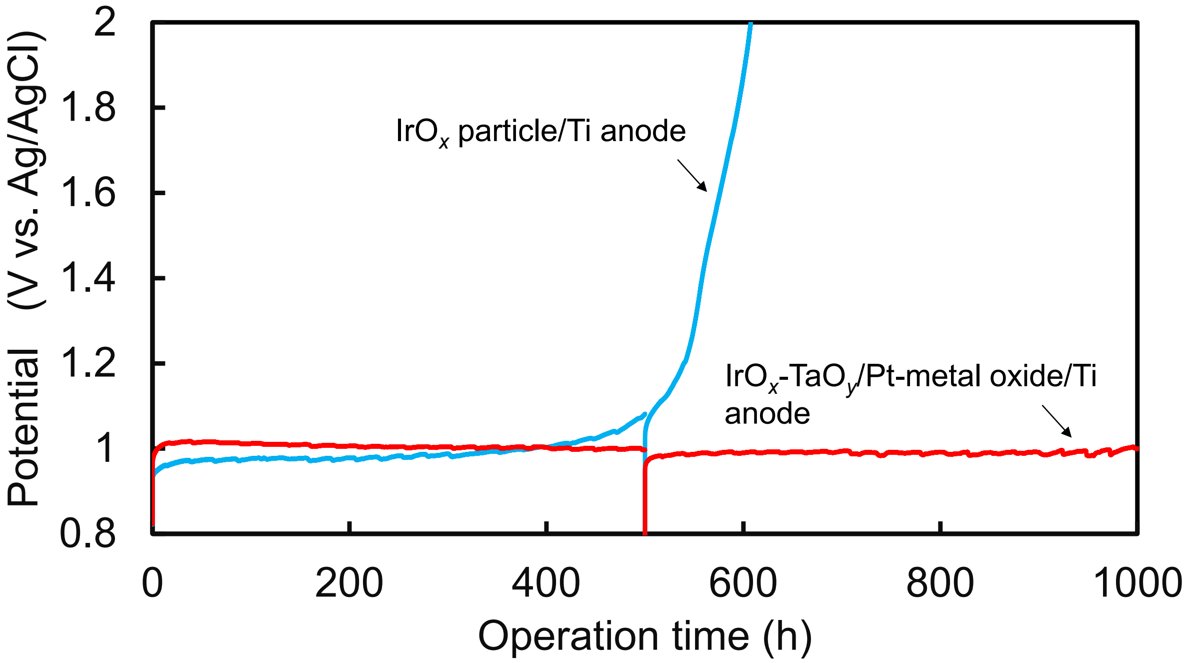

Figure S5. Comparison between the previous IrO_x particle/Ti anode and new IrO_x-TaO_y/Pt-metal oxide/Ti anode of 1 cm^2 in size. Voltage–time (*V*–*t*) measurements were conducted at a constant current of 1 mA. During the first 300 h, the potential of the new anode was slightly higher than that of the IrO_x/Ti anode, indicating slightly lower water-oxidation activity. Subsequently, the potential of the IrO_x/Ti anode gradually increased, followed by a rapid increase starting at 500 h with its color changing from dark blue to transparent light blue. These changes were caused by the detachment of the IrO_x particles. In contrast, the potential of the new anode was extremely stable up to 1000 h. Consequently, the new IrO_x-TaO_y/Pt-metal oxide/Ti anodes were adopted for preparing the newly developed Type-A EC reactors.

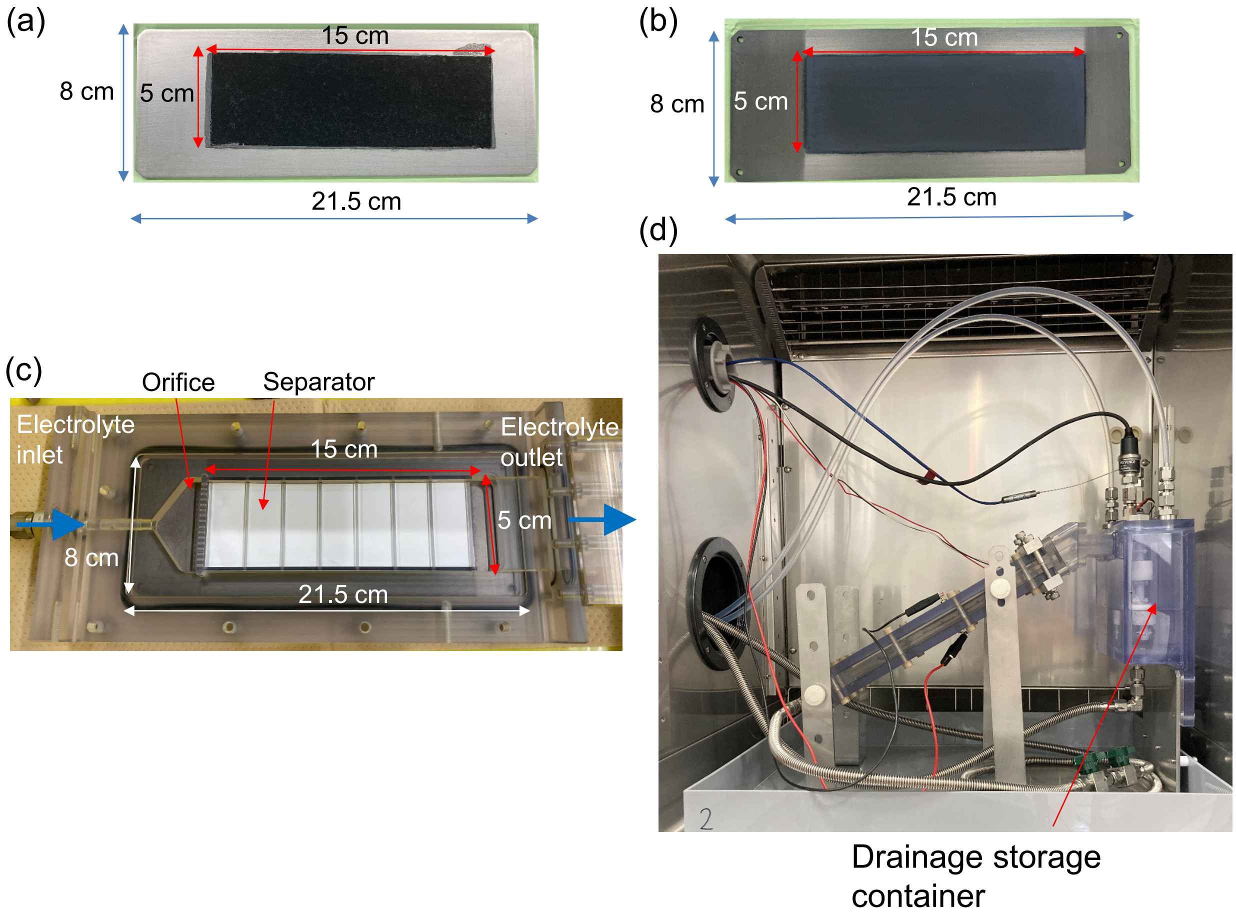

Figure S6. Appearance of the 75 cm^2-sized Type-A cathode, anode, and EC reactor. (a) Cathode using the RuCP/MWCNTs/CS/G3 graphite adhesive/Ti-plate with the UV-ozone treatment, Sol. 5 including the large amount of the pyrrole derivative equipped with amino group, and post-loading. (b) Anode using the IrO_x-TaO_y/Pt-metal oxide/Ti-plate. (c) EC reactor housing and electrolyte flow channel equipped with the orifice plate and separator. (d) EC reactor installed at 30 °.

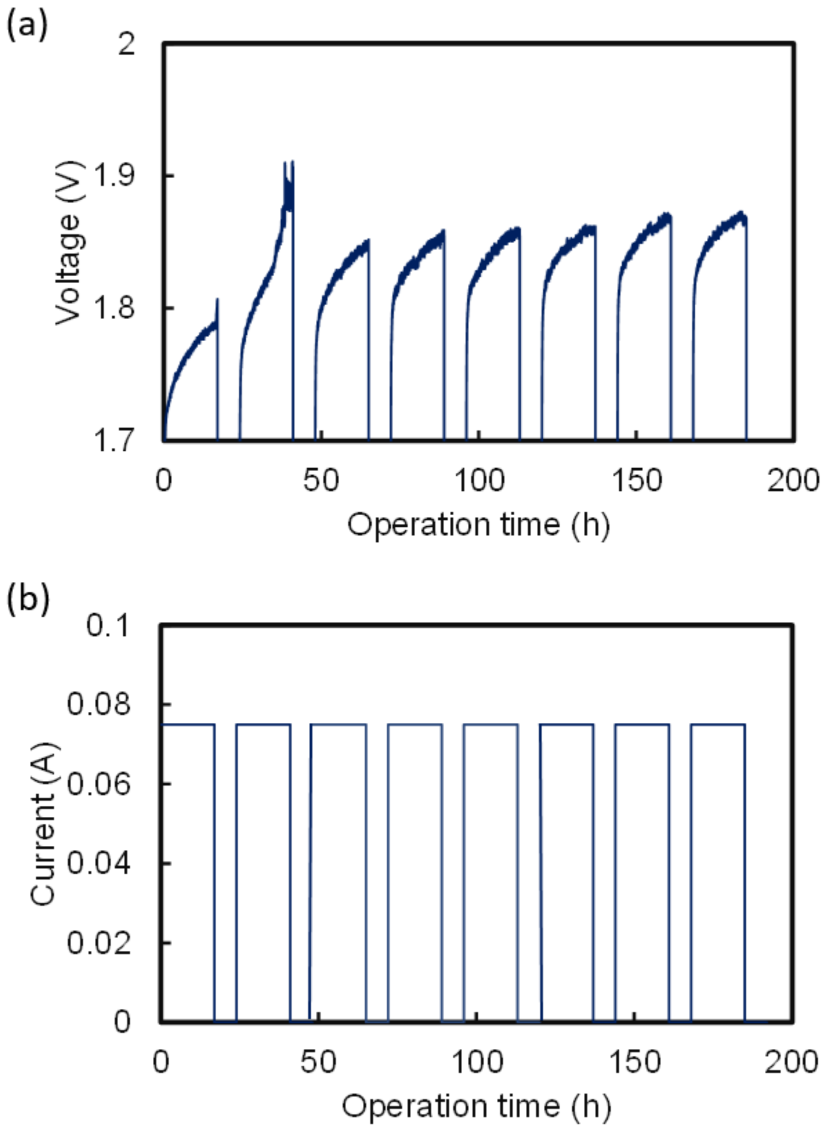

Figure S7. (a) Voltage and (b) current during the long-term durability test on the 75 cm^2-sized Type-A EC reactor in combination with the IrO_x-TaO_y/Pt-metal oxide/Ti anode and RuCP/MWCNTs/CS/G3 adhesive/Ti cathode with the UV-ozone treatment, Sol. 5 including the large amount of the pyrrole derivative equipped with amino group, and post-loading, during the long-term durability tests. The intermittent operation at a current of 75 mA for 17 h and at 0 mA for 7 h alternately in a day was adopted.

Table S3. Amounts of Ru detached from the carbon supports and dissolved in the electrolyte of the 75 cm^2-sized Type-A EC reactor during the long-term intermittently operating durability test, measured by ICP-MS. The reactor adopted the IrO_x-TaO_y/Pt-metal oxide/Ti anode and RuCP/MWCNTs/CS/G3 adhesive/Ti cathode with the UV-ozone treatment, Sol. 5 including the large amount of the pyrrole derivative equipped with amino group, and post-loading. The electrolyte was replaced with a fresh one approximately every 100 h. The Ir concentrations were lower than the detection limit of 5 ng/mL and hence the detachment rates were smaller than 0.66%/100 h. The concentration of "before" was the value measured immediately after the electrode was immersed in the electrolyte.

Time of sampling	Concentration of Ru (ng/mL)	Detachment rate of Ru (%/100 h)
Before	10	–
After 198 h	10	0.26
After 544 h	10	0.26
After 1139 h	10	0.26
After 1479 h	20	0.52

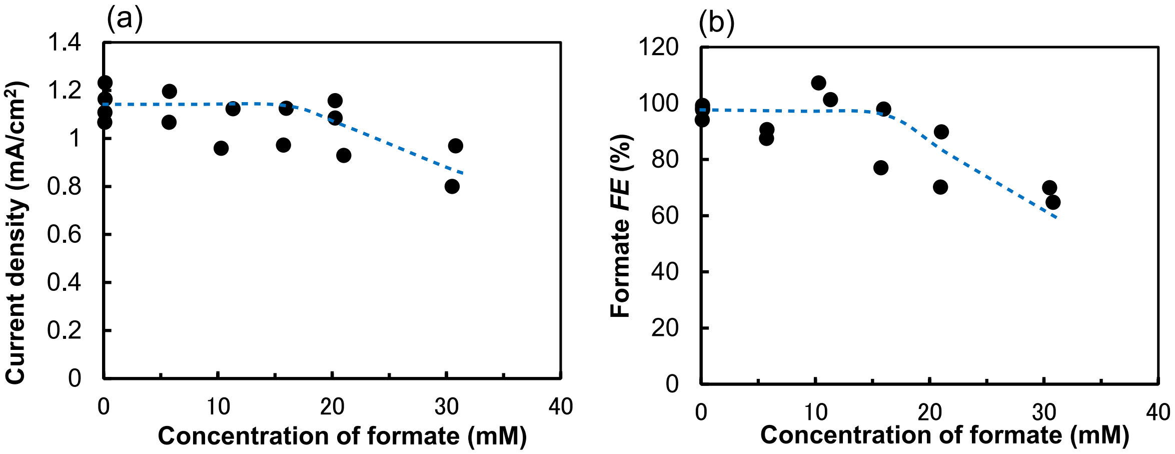

Figure S8. Impacts of the formate concentration dissolved in the electrolyte on the formate-production performance of the 1cm^2-sized Type-B cathodes. Current–time (*I*–*t*) measurements were conducted for 1 h at a constant potential of −1.2 V vs. Hg/Hg_2SO_4. 6, 10, 16, 21, 30 mM of formic acid were added into the 0.4 M KPi electrolytes before starting the measurements, and no addition for comparison. Few impacts were observed up to 15 mM, followed by gradual decreases in both the current density and formate *FE*. Therefore, the electrolyte was periodically replaced with a fresh one before the formate concentration reached 20 mM during the long-term durability tests.

Table S4. Orifice patterns designed for the 75 cm^2-sized EC reactors. The ratio of the total area of the orifices to the cross-sectional area of the flow channel in the cathode-side (orifice area ratio), as well as the arrangements of the orifices, were changed; See also Figure S9. Pattern No. 4 realized the most uniform flow, and consequently it was adopted for the 75 cm^2-sized Type-A EC reactors.

**Pattern**	**No. 1**	**No. 2**	**No. 3**	**No. 4**	**No. 5**
Orifice area ratio (%)	4.36	1.84	7.38	4.36	4.36
Arrangement	Equidistant	Equidistant	Equidistant	Uneven spacing	Uneven spacing
Standard deviation of flow velocity	7.47×10^{-4}	9.18×10^{-4}	6.83×10^{-4}	6.72×10^{-4}	1.14×10^{-3}

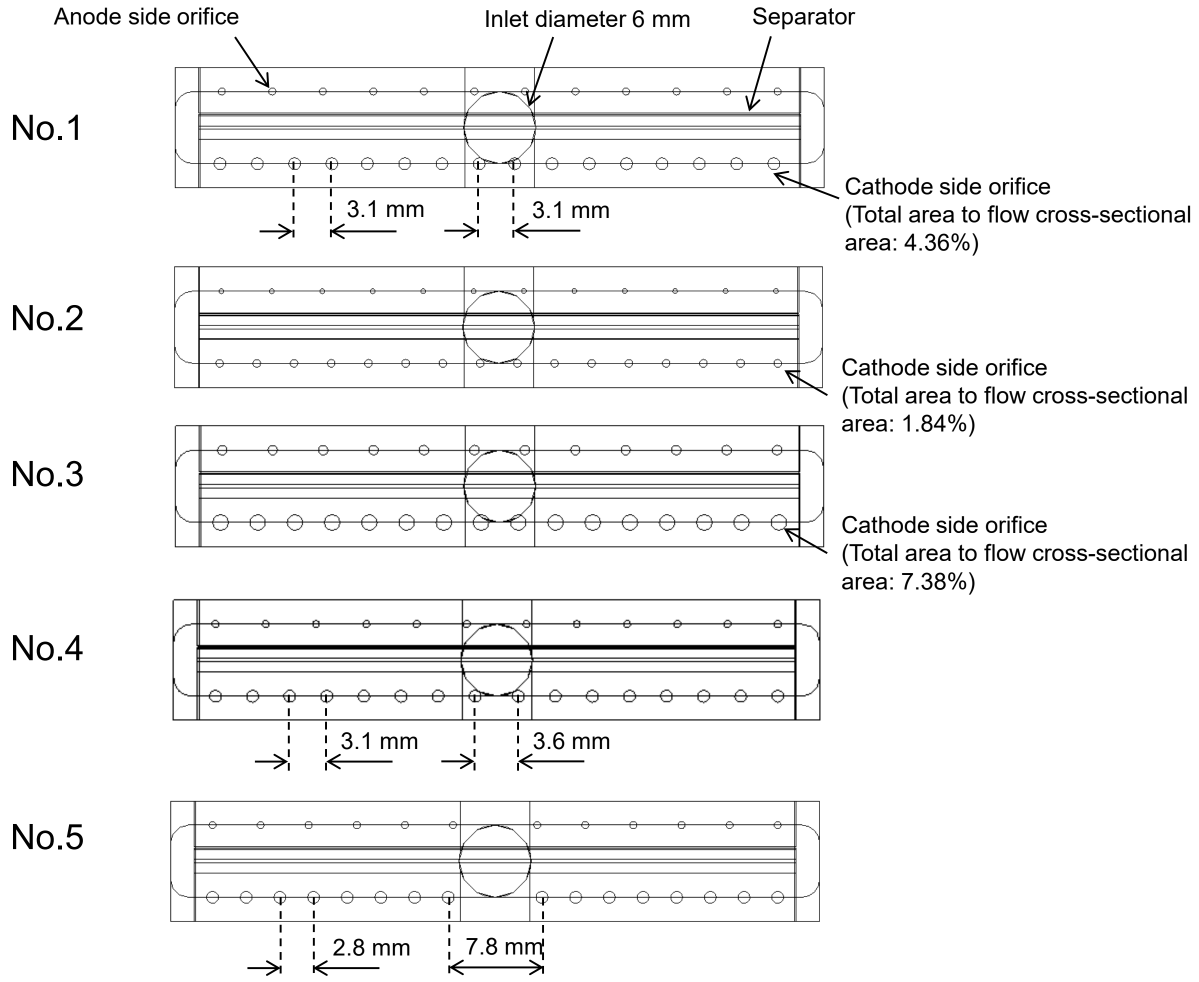

Figure S9. Orifice plates of No. 1−No. 5 viewed from the electrolyte-flow direction. See also Table S3.

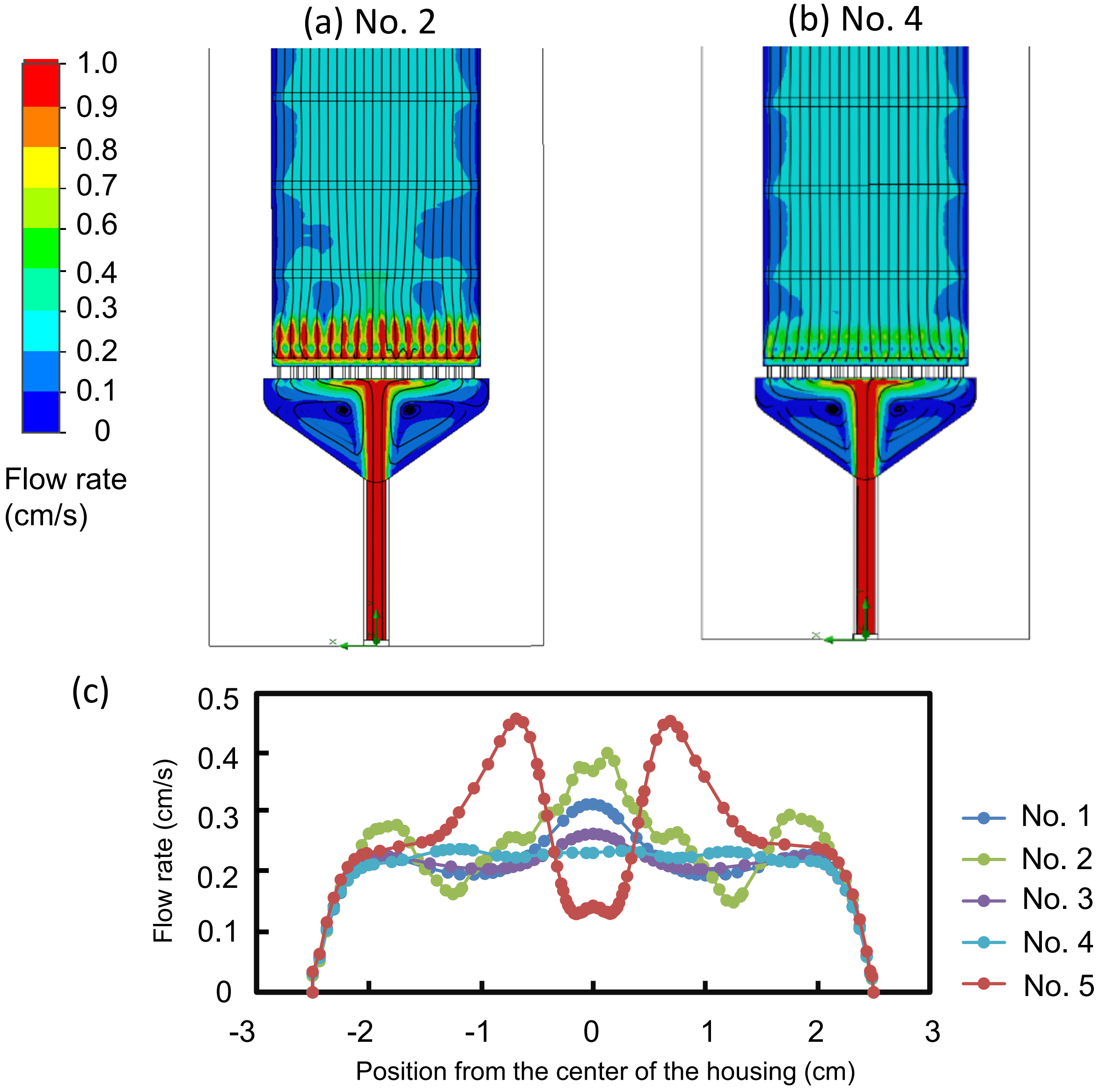

Figure S10. Simulated results of the flow rate distributions. Two-dimensional distributions for (a) No. 2 and (b) No. 4. (c) Comparison among the five orifice patterns evaluated at 15 mm from the orifice plates. Pattern No. 4 realized the most uniform flow, and consequently it was adopted for the 75 cm^2-sized Type-A EC reactors. See also Table S3.
